# Brain responses to different action observation paradigms and assessing transferable cross-paradigm decoding

**DOI:** 10.1186/s12984-026-01946-3

**Published:** 2026-03-14

**Authors:** Guiyu Hu, Hongmei Tang, Fukang Zeng, Xingyu Wen, Wensheng Hou, Xin Zhang

**Affiliations:** 1 The College of Bioengineering, Chongqing University, Chongqing, 400044 China; 2 The Key Laboratory of Biorheological Science and Technology, Ministry of Education, Chongqing University, Chongqing, 400044 China

**Keywords:** Brain-computer interface, Action observation, Steady state motion visual evoked potentials, Electroencephalogram responses, Cross-AO paradigms

## Abstract

**Background:**

Action observation-based brain-computer interface (AO-BCI) is a novel technology for stroke rehabilitation. Since various AO paradigms are employed in the rehabilitation of different limb movements, a limited training dataset can compromise recognition accuracy. Thus, this study aimed to analyze the brain responses to various AO paradigms systematically and assess the BCI recognition performance across AO paradigms for the first time.

**Methods:**

On the basis of a frame rate reduction method, three AO paradigms, each containing four actions, were designed: the right-hand task-driven (RHTD) paradigm, the right-hand non-task-driven (RHNTD) paradigm, and the left-hand task-driven (LHTD) paradigm. EEG data were collected from 19 participants under the visual feedback condition and the soft robotic glove feedback condition. Task discriminative component analysis (TDCA) was utilized to perform online target detection. EEG responses were analyzed. Moreover, four training schemes, including target session (TS) data, source session data, a combination and transfer with least squares transformation, were developed to construct spatial filters in TDCA to assess the transferability of cross-paradigm decoding in AO-BCI.

**Results:**

The three designed AO paradigms elicited steady state motion visual evoke potentials (SSMVEP) in the occipital cortex and induced event-related desynchronization (ERD) in the sensorimotor cortex. The RHTD paradigm induced stronger ERD than the other two paradigms. In addition to EEG responses, the recognition accuracy of the SSMVEP under the RHNTD paradigm (85.20%) was significantly greater than that under the RHTD paradigm (72.37%) and the LHTD paradigm (77.50%). Furthermore, when combing EEG from the mirrored stimulus and TS data, the recognition accuracy increased to 77.50% (RHTD) and 80% (LHTD) respectively. However, when EEG data from observing the AO paradigm, whether task-driven or not, were directly used for training or combed with TS data, the recognition accuracy decreased.

**Conclusion:**

This study revealed that the content of AO paradigms affects the amplitude of the evoked SSMVEP and ERD. Furthermore, EEG data elicited by the mirrored stimulus can be directly utilized in cross-paradigm training to increase recognition accuracy, whereas data from observing paradigms involving task-driven or not require further development of calibration methods to be effectively applied in cross-paradigm decoding.

## Background

The rapid shift toward aging societies is driving up stroke rates, making it a leading cause of public health burden. Cerebral ischemia frequently leads to motor impairment, with approximately 80% of survivors exhibiting upper-limb paresis and over 60% of chronic patients experiencing persistent deficits that compromise independence. Effective upper limb motor function rehabilitation is an essential means for stroke patients to return to a fulfilling life. Hand function recovery requires the integration of sensory, motor, and cognitive functions, making it more challenging and complex compared to rehabilitation training for other parts of the body [1, 2]. Neural plasticity enables the brain to achieve functional compensation post-stroke by reorganizing unaffected regions [3]. While traditional rehabilitation methods have a certain degree of efficacy, their rehabilitation effectiveness is significantly limited because they rely on passive training modes [4].

Compared to traditional passive training approaches, brain-computer interface (BCI) [5, 6] enables stroke patients to actively engage in motor rehabilitation, thereby promoting neural plasticity and improving rehabilitation outcomes [7]. The action observation (AO) paradigm, one of the BCI paradigms, is based on the mirror neuron system (MNS) and can activate the sensorimotor cortex [8]. Mirror neurons were first discovered in monkeys and later confirmed in humans [9]. Through the MNS, the AO can activate the sensorimotor cortex and generate electroencephalogram (EEG) signals similar to those produced during actual movement execution or motor imagery. Compared with motor imagery (MI), the visual stimuli in AO are more readily executed and elicit stronger brain responses [10]. Clinical studies have confirmed that AO can effectively improve neurological and motor dysfunction [11, 12]. In practical applications, AO is often combined with MI to form the AO-MI task, which enhances action representation and event-related desynchronization (ERD), thereby improving motor decoding efficiency and facilitating motor function rehabilitation [13–15]. A recent study also showed that both ERD intensity and classification accuracy of MI primed by AO were more significant than MI instructed by arrows [16].

Action observation-based brain-computer interface (AO-BCI), which are designed on the basis of AO treatment therapy, can be used for motor function rehabilitation and accelerates and improves functional recovery in stroke patients [17]. In AO-BCI, patients observe actions via video or live demonstration and vividly imagine performing the same actions themselves. This process also engages neuroplastic mechanisms, thereby promoting the subsequent execution of related daily activities. It activates neural networks associated with the observed behavior and is closely linked to the mirror neuron system. Furthermore, AO-BCI can simultaneously induce steady-state motion visual evoked potential (SSMVEP) in the occipital cortex and sensory motor rhythm (SMR) in the sensorimotor cortex [15]. This unique dual-brain-region activation characteristic holds significant application potential in stroke rehabilitation.

The AO paradigm encompasses a wide range of action types, and different actions elicit significantly distinct brain activation patterns, with their neural effects modulated by the functional architecture of the MNS. Research indicates that MNS activation is modulated by the specificity of the observed action [18]. Moreover, the lateralization effect in the AO is also critical. The design of mirrored limb conditions was informed by the findings of Gandi DB et al. on mirror neurons [19], which suggested that observing the contralateral movements of a healthy limb can activate the sensorimotor cortex of the affected side, thereby promoting its functional reorganization through transhemispheric mirror neuron pathways. Several studies have confirmed that training the unaffected limb can significantly improve the motor function of the affected limb [20, 21]. Lee et al. further demonstrated that task-driven MI (e.g., grasping an object) induces stronger µ/β rhythm ERD than non-task-driven MI, thereby enhancing motor decoding efficiency and rehabilitation outcomes [22]. However, current AO research has not yet examined the regulatory effects of task-driven mechanisms on the coordinated activation of brain regions. Therefore, this study systematically analyzed the differences in brain activation patterns induced by three AO paradigms which were designed on the basis of task-driven demands and the mirrored stimulus.

In addition, the core challenge for practical BCI applications lies in achieving strong generalizability. Existing cross-domain BCI research has extensively explored scenarios such as cross-day, cross-subject, and cross-device conditions, primarily focusing on methods such as transfer learning, domain adaptation, and domain generalization to enhance model generalizability [23–25]. However, limited attention has been given to the transferable cross-paradigm decoding. Patients with hand motor dysfunction undergo training for various hand movements, including individual finger flexion/extension, mirrored movement, finger opposition, thumb opposition, tapping, grasping, and so on. AO-based BCIs represent a potential pathway for hand motor function recovery. Expanding the number of movement categories substantially increases the difficulty of decoding the multiclass movement intents, which typically leads to performance degradation. Cross‑AO‑paradigm decoding allows data from existing paradigms to support new training content without redundant data collection, while preserving decoding performance. Since SSMVEP in the occipital cortex induced by AO might be transferable, and different actions suit different rehabilitation needs, studying cross-paradigm decoding performance in AO-BCI is essential.

This study investigated differences in brain responses elicited by different AO paradigms and their impact on recognition accuracy, with a focus on the transferable cross-AO paradigms decoding. Therefore, the foundation for developing data calibration across AO paradigm decoding is established, expanding the data sources for training decoding models in practical AO-BCI applications. Two key dimensions were examined, the mirrored stimulus (left versus right-handed movements) and the task-driven actions (task-driven vs. non-task-driven), to explore their effects on brain responses and across AO paradigm decoding. Compared with traditional AO tasks, a soft robotic glove was designed to provide action feedback, enabling combined active-passive hand rehabilitation. EEG features from the occipital cortex and the sensorimotor cortex were extracted to compare AO-specific activation patterns. Task discrimination component analysis (TDCA) [26] was employed to construct spatial filters based on EEG data from the occipital cortex for decoding, elucidating the influence of AO paradigms on AO-BCI decoding. For cross-paradigm decoding, the test data paradigm was labeled the target session (TS), and the others were labeled the source sessions (SS). Four TDCA spatial filter training schemes were designed to assess the transferability of cross-paradigm decoding in AO-BCI.

## Methods

### Subjects

This study recruited 19 healthy participants. The average age of the participants was 23 ± 2.81 years. The sex distribution included 5 females and 14 males. With respect to experience with BCI devices, 12 participants were naive participants, while the remaining 7 participants had limited prior experience (≤ 3 times). All the participants were right-handed. Prior to the experiment, the research team provided a comprehensive description, covering duration, operational steps, and potential discomfort. After fully understanding the experimental content, all the participants voluntarily signed written informed consent forms, clearly acknowledging their rights and obligations as participants, as well as their right to withdraw from the experiment at any time. The ethical review for this study was approved by the Ethics Committee of the People’s Hospital of Shapingba District, Chongqing City, China (Approval Number: KY202412).

### EEG acquisition system

In this study, a 32-channel EEG system (ANT neuro, eego mylab) was used to record EEG data. Electrodes were arranged according to the extended International 10/20 system [27, 28], with specific locations including: Fp1, Fpz, Fp2, F7, F3, Fz, F4, F8, FC5, FC1, FC2, FC6, T7, C3, Cz, C4, T8, CP5, CP1, CP2, CP6, P3, Pz, P4, P7, M1, POz, M2, P8, O1, Oz, and O2. The reference electrode was placed on CPz, and the ground electrode was placed on AFz. The experiment employed a 50 Hz hardware notch filter to preprocess the acquired signals, thereby reducing the impact of power frequency interference on the signals and ensuring the quality of the acquired EEG signals. The sampling rate of the EEG signals in this study was 500 Hz.

In the online experiment, MATLAB was used to record EEG data collected by the EEG acquisition system, and the experimental paradigm, decoding device, online decoding algorithm, event timestamps (including the start and end times of each trial), and corresponding true labels were recorded synchronously. Corresponding data files and event files were generated to enable researchers to match EEG signals accurately with specific experimental events during the offline analysis phase, thereby enabling a deeper exploration of neural response patterns in the brain across different task phases.

### AO paradigms design and experimental procedure

**Fig. 1:**
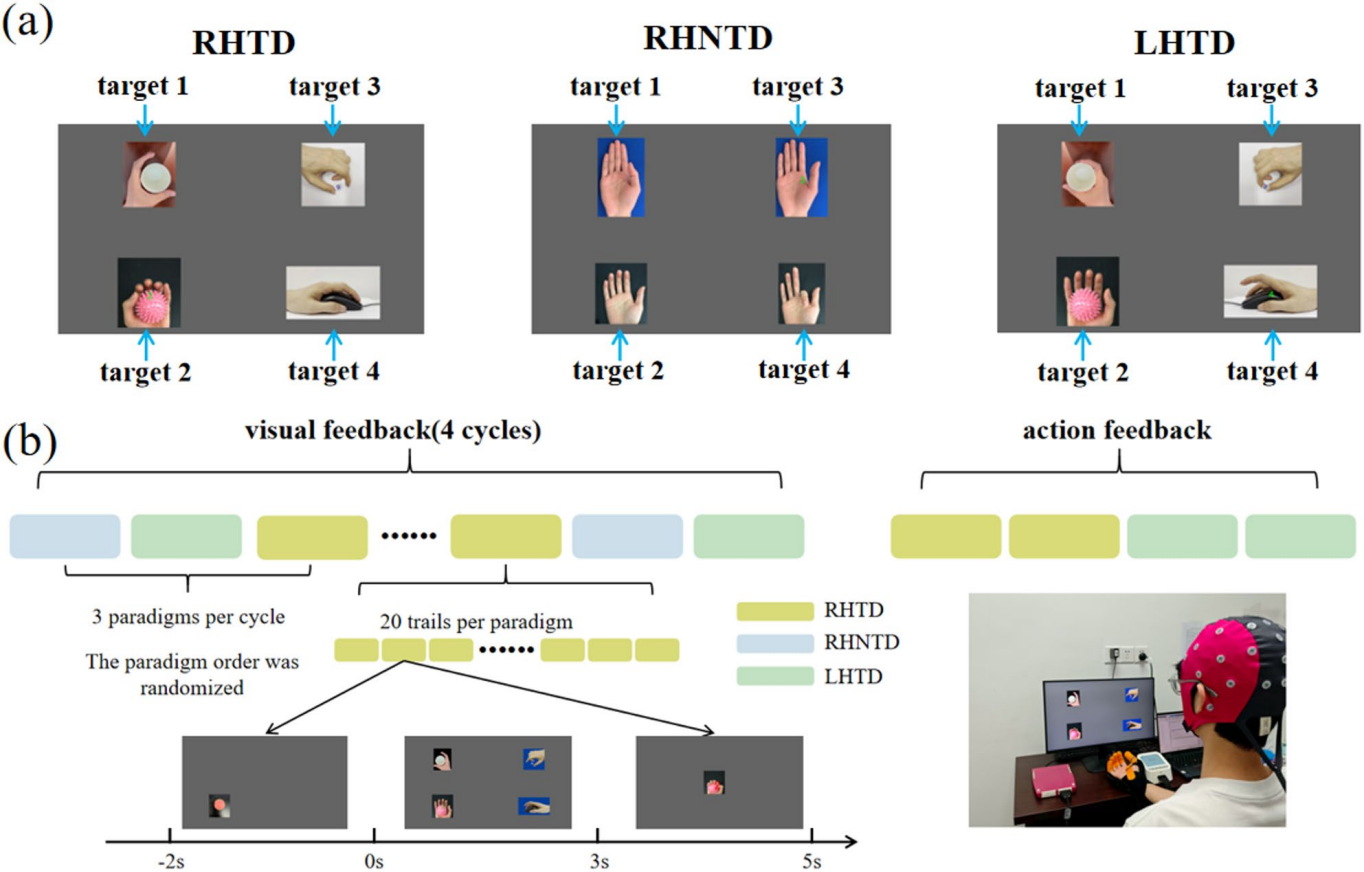
Design of the AO paradigms and the experimental procedure **a** Three AO paradigms. **b** Experimental procedure diagram, in which visual feedback collects all paradigm data, and action feedback collects data from the RHTD and LHTD paradigms

This study designed three hand AO stimulation paradigms, each containing four specifics actions (four targets). This study adopted a frame rate reduction method [29] to ensure that each action image lasted for *N*/60 seconds, and the action movement frequency was precisely set to 60/(*N_AO*×*N*), where *N_AO* represents the number of images captured during the action cycle. The action movement frequency of the same position actions in the three AO paradigms was kept consistent, and the frame rates (*N* = 13, 11, 8, 9) of the four actions were set to 4.6154 Hz, 5.4545 Hz, 7.5000 Hz, and 6.6667 Hz, respectively, providing standardized stimulation conditions for subsequent cross-AO paradigms decoding studies. The specific details are shown in Fig. 1(a). The four stimulus actions (target 1, 2, 3, and 4) of the right-hand task-driven (RHTD) paradigm are grasping a paper cup, grasping a small ball, pinching a dice with the thumb and index finger, and tapping the mouse with the index finger. The four stimulus actions (target 1, 2, 3, and 4) in the right-hand no-task-driven (RHNTD) paradigm are thumb adduction, grasping, pinching with the thumb and index finger, and bending the index finger. The stimulus actions (target 1, 2, 3, and 4) in the left-hand task-driven (LHTD) paradigm are identical to those in the RHTD paradigm, except that they are performed with the opposite hand.

Prior to the online data collection experiment in this study, the participants were instructed to remain relaxed, sit with their hands apart at the arm’s length from a 24-inch full-color screen, and concentrate on the AO task, and mentally simulate the observed actions synchronously (i.e., perform an MI task). The online collection process is shown in Fig. 1(b). The online collection experiment was divided into two collection stages based on the form of feedback: visual feedback and action feedback. Visual feedback presented the decoding results of the stimulus to the subjects through the screen, whereas action feedback determined whether Bluetooth would send an action feedback signal to the soft robotic glove to drive the subjects’ hands to perform the predesigned corresponding actions based on the correctness of the subjects’ real-time decoding results. In the visual feedback stage, the three AO paradigms are presented alternately with a total of four runs for each paradigm. In the action feedback stage, the RHTD paradigm and LHTD paradigm are presented alternately with a total of two runs for each paradigm. The four actions in each paradigm are presented randomly five times in one run, i.e., twenty trials in one run. Each trial contains three phases: (1) The cue phase: Two seconds before the stimulus, the screen displays the target stimulus action location to help participants focus their attention. (2) The stimulus phase: The target stimulus action lasts for 3 s, during which participants direct their attention to observe the action and engage in motor imagery. (3) The feedback phase: Real-time display of decoding results with sustained presentation (feedback by visual/action: 2s/5s). During action feedback, when decoding results were correct, the soft robotic glove guided the subject’s hand to perform a pre-designed movement. Subjects could relax during this phase and prepare for the next round. Rest periods are scheduled between different AO paradigm stimulation trials to ensure subjects remain alert. The run order was randomized. The participants were instructed to focus on only one stimulus target at one trial. In summary, a complete online data collection experiment will present 16 AO-paradigm runs: 12 with visual feedback and 4 with action feedback.

### EEG feature extraction and analysis in different brain regions

The acquired EEG data in the occipital cortex and the sensorimotor cortex were analyzed. First, the open-source toolbox EEGlab (version 2024.2) running under MATLAB was used to import the data. A FIR filter was then applied for bandpass filtering at 3–40 Hz to eliminate high-frequency and low-frequency noise. According to the event timestamps, relevant data were precisely selected from continuous EEG signals for segmentation. The moment of stimulus onset was set as the baseline (‘0’ moment). Baseline correction was applied using a window from 800 ms before to 0 ms relative to this onset. Subsequently, artifact removal was performed using Independent Component Analysis (ICA) implemented in the EEGlab toolbox (v2024.2). The ICLabel plugin was employed to automatically classify all independent components; components identified as ocular or muscular artifacts with a confidence score > 80% were removed. Finally, manual screening was conducted to exclude bad segments based on an objective amplitude criterion, whereby any segment exceeding 200 µV was rejected, resulting in an average exclusion rate of approximately 6.8%. This standardized preprocessing pipeline establishes a consistent foundation for the subsequent analysis of regional features and paradigm differences.

(1) Occipital cortex: This study focused on the response of the occipital cortex to stimuli in the time domain and frequency domain. The research indicator was based on the EEG signal values of the Oz channel, which were converted from the time domain signal to the frequency domain signal using the discrete Fourier transform to reveal the amplitude and phase information of different frequency components in the signal.

(2) Sensorimotor cortex: To improve feature space specificity, this study used the Laplace operator to process C3/C4 channel data. The Laplacian value for each target electrode *i* (e.g., C3/C4) is calculated as the difference between its original potential and the average potential of its six neighboring electrodes (FC5, FC1, T7, CP5, CP1, Cz/FC6, FC2, T8, CP6, CP2, Cz):

1 $${L_i}={V_i} - \frac{1}{N}\sum\limits_{{j \in {\Omega _i}}} {{V_j}} $$

Where *L*_*i*_ is the Laplace value of electrode *i* (C3/C4), *V*_*i*_ is the original potential of electrode *i*, *V*_*j*_ is the neighborhood set of electrode *j*, and *N* is the number of neighborhood electrodes, which is 6. This operation can effectively reduce broadband EEG noise and improve reliability [30].

Subsequently, the event-related spectral power (ERSP) was computed, which represents the average change in event-related spectral power relative to the baseline at each time point and frequency during the experiment [31–33]. The ERSP affords researchers with the ability to conduct in-depth analyses of changes in specific frequency components, thereby enabling more precise revelations of brain activity patterns during the execution of different cognitive and sensory tasks. Importantly, the ERSP not only illustrates event-related average potential changes but also reveals the dynamic evolution of neural activity. The laterality index (LI) is a standardized metric used to quantify the asymmetry of brain function between the left and right hemispheres. By comparing the activation intensity of the left and right hemispheres during a specific task, it indicates the degree of lateralization of neural activity, i.e., which hemisphere predominates during a given task as reflected in EEG parameters. LI is widely applied in investigating the neural mechanisms underlying cognitive functions such as motor control, language processing, and attention, particularly in stroke rehabilitation, preoperative epilepsy assessment, and BCI. Studies have shown that LI ranges from − 1 to 1, representing complete lateralization towards the ipsilateral or contralateral hemisphere, respectively [34]. Therefore, this study analyzed EEG data from the motor regions C3 and C4 channels using LI based on ERSP, with a selected frequency band range of 5–30 Hz and the α (8–12) and β (13–30) bands.

2 $$LI=\frac{{{\mathrm{ER}}{{\mathrm{D}}_{C3}} - {\mathrm{ER}}{{\mathrm{D}}_{C4}}}}{{{\mathrm{ER}}{{\mathrm{D}}_{C3}}+{\mathrm{ER}}{{\mathrm{D}}_{C4}}}}$$

The *ERD*_*C3*_*/ERD*_*C4*_ represent the ERD of channel C3 and the ERD of channel C4, respectively.

### Online target recognition method

This study implements the TDCA algorithm, proposed by Gao Xiaorong’s team in 2021 [26], performing online decoding within the EEG data from the occipital cortex under the AO paradigms. EEG data from electrodes P7, P3, P4, P8, POz, O1, Oz, and O2 were selected. The 3–30 Hz bandpass Butterworth filter was applied to the EEG data from the stimulus phase. The algorithm exploits the temporal structure of EEG signals and, as a discriminative model, learns shared spatial filters across all stimulus frequencies, thereby eliminating redundant computations.

In detail, TDCA applies a time-delayed stacking on each trial EEG to augment dimensionality, and uses QR decomposition to construct orthogonal projections that preserve stimulus-frequency components. the number of time-delayed stacks in the TDCA module is set to 0. No filter bank is employed in this study. The sine-cosine reference signal *Y*_*i*_ was utilized for the QR decomposition.

3 $${{\mathrm{Y}}_{\mathrm{i}}}{\mathrm{=}}\left[ {\begin{array}{*{20}{c}} {\begin{array}{*{20}{c}} {{\mathrm{sin(2}}\pi {{\mathrm{f}}_{\mathrm{i}}}{{\mathrm{t}}^{\mathrm{T}}}{\mathrm{)}}} \\ {{\mathrm{cos(2}}\pi {{\mathrm{f}}_{\mathrm{i}}}{{\mathrm{t}}^{\mathrm{T}}}{\mathrm{)}}} \end{array}} \\ {\begin{array}{*{20}{c}} {{\mathrm{sin(4}}\pi {{\mathrm{f}}_{\mathrm{i}}}{{\mathrm{t}}^{\mathrm{T}}}{\mathrm{)}}} \\ {{\mathrm{cos(4}}\pi {{\mathrm{f}}_{\mathrm{i}}}{{\mathrm{t}}^{\mathrm{T}}}{\mathrm{)}}} \end{array}} \end{array}} \right]{\mathrm{,t=}}{\left[ {\frac{{\mathrm{1}}}{{{{\mathrm{f}}_{\mathrm{s}}}}}{\mathrm{,}} \ldots {\mathrm{,}}\frac{{{{\mathrm{N}}_{\mathrm{p}}}}}{{{{\mathrm{f}}_{\mathrm{s}}}}}} \right]^{\mathrm{T}}}$$

where *f*_*s*_ denotes the sampling rate, and *N*_*p*_ is the number of sampling points. In this study, *f*_*s*_=500 Hz, *N*_*p*_=1500.

Subsequently, the original and projection data are combined to form composite features, and the optimal spatial filter *W* is derived under Fisher’s criterion.

Finally, features are extracted via *W*, and labels are predicted through template matching. The optimal spatial filter *W* is obtained by maximizing the intergroup differences and minimizing the intragroup differences.

4 $$\mathop {\hbox{max} }\limits_{W} \frac{{tr({W^T}{S_b}W)}}{{tr({W^T}{S_w}W)}}$$

During the test stage, identical augmentation procedures are applied to ensure structural consistency with the training data. Then test sample ${\mathbf{X}}_{test}^{i}$ of $i$th class is mapped to the discriminant subspace through the trained projection matrix:

5 $${\mathrm{Z}}_{{{\mathrm{test}}}}^{{\mathrm{i}}}{\mathrm{=(}}{{\mathrm{W}}^{\mathrm{*}}}{{\mathrm{)}}^{\mathrm{T}}} \cdot {\mathrm{X}}_{{{\mathrm{test}}}}^{{\mathrm{i}}}$$

The correlation coefficient ${\rho}^{i}$ between each projected test feature ${\mathbf{Z}}_{test}^{i}$ and its corresponding class template ${\boldsymbol{\mu}}^{i}$ was then computed.

The corresponding template of each class $i$ is obtained by averaging the projected features of all training trials:

6 $${{{\mu}}^{\mathrm{i}}}{\mathrm{=}}\frac{{\mathrm{1}}}{{\mathrm{K}}}\,\sum\nolimits_{{K=1}}^{K} {{{{\mathrm{(}}{{\mathrm{W}}^{\mathrm{*}}}{\mathrm{)}}}^{\mathrm{T}}} \cdot {\mathrm{X}}_{{{\mathrm{train}}}}^{{{\mathrm{k,i}}}}} $$

where $K$ is the total number of training trials for class $i$.

The class ${i}^{*}$ with the maximum correlation coefficient is assigned as the final predicted result.

### Design of the decoding of cross-AO paradigms

**Fig. 2:**
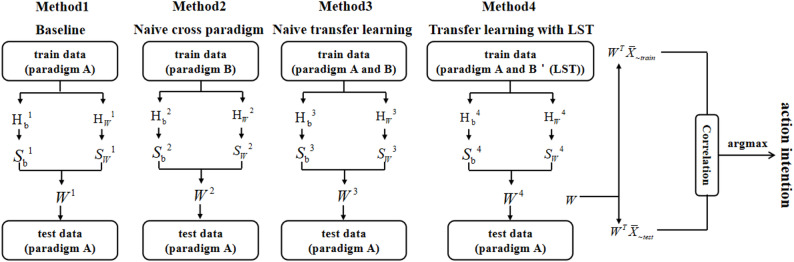
Design of the cross-AO paradigms decoding scheme

This study applied TDCA to decode and analyze the EEG segment comprising 1,500 sampling points (sampling rate × data length) during the stimulus phase under the visual feedback condition. In the cross-AO paradigms decoding scheme, four training set configurations were designed to construct spatial filters, as illustrated in Fig. 2.

(1) Baseline (Method 1): Training data and testing data are from the same AO paradigm, i.e., no cross-AO paradigms decoding is performed, to explore the target identification accuracy of TDCA across different AO paradigms.

(2) Naive cross paradigm (Method 2): Training data are from the SS paradigm, and testing data is from the TS paradigm, thereby examining the influence of SS data on TS paradigm decoding.

(3) Naive transfer learning (Method 3): The SS paradigm data are directly concatenated with the TS paradigm training data, and the merged training set is used for TS paradigm testing. This approach omits explicit modelling of inter-domain distributional differences, serving only to evaluate the effect of simple concatenation on TS decoding.

(4) Transfer with the least squares transformation (LST) (Method 4): The LST [35] was proposed by UCSD Chiang et al. It is a generalized transfer framework for template matching that derives for the closed-form transformation matrix P by minimizing the covariance difference between SS and TS, thereby mapping the SS to the TS space. This facilitates accuracy improvements across subjects, devices, and days with minimal calibration data. The analytical solution for the transformation matrix P is as follows:

7 $$P=x{\tilde {x}^T}{(\tilde {x}{\tilde {x}^T})^{ - 1}}$$

The LST is employed to project the SS data onto the TS distribution, which is then merged with TS training data for TS AO paradigm testing. By quantifying the calibration efficacy of this generalized transfer strategy across AO paradigms, further development of dedicated data adaptation methods is necessary.

Besides, a fourfold cross-validation scheme was used to evaluate the target recognition accuracy. One run’s data were retained as the validation data for testing, and the other three runs’ data were used as training data. There was no overlapping part in either the training or test subsets. The cross-validation process was then repeated four times, with each of the four runs’ data used exactly once as the validation data.

### Statistical methods

Statistical analyses were conducted at a significance level of ${\upalpha}$=0.05. A mixed-effect analysis of variance (ANOVA) was employed to compare differences in brain responses across different AO paradigms and in recognition accuracy under different AO paradigms and different data lengths, with Fisher’s post-hoc test used to assess significance. One-way ANOVA followed by Fisher’s post-hoc test was applied to assess the significance of the differences in recognition accuracy across the AO paradigms. A paired t-test was employed to assess the difference in decoding effectiveness between action feedback provided by the soft robotic glove and visual feedback. To control the family-wise error rate in multiple comparisons, p from post-hoc tests were adjusted using the Bonferroni correction. Adjusted p was reported as such. For single primary hypothesis tests (e.g., paired t-tests on feedback conditions), the significance threshold was set at $\alpha$ = 0.05.

## Result

### Analysis of occipital cortex characteristics

**Fig. 3:**
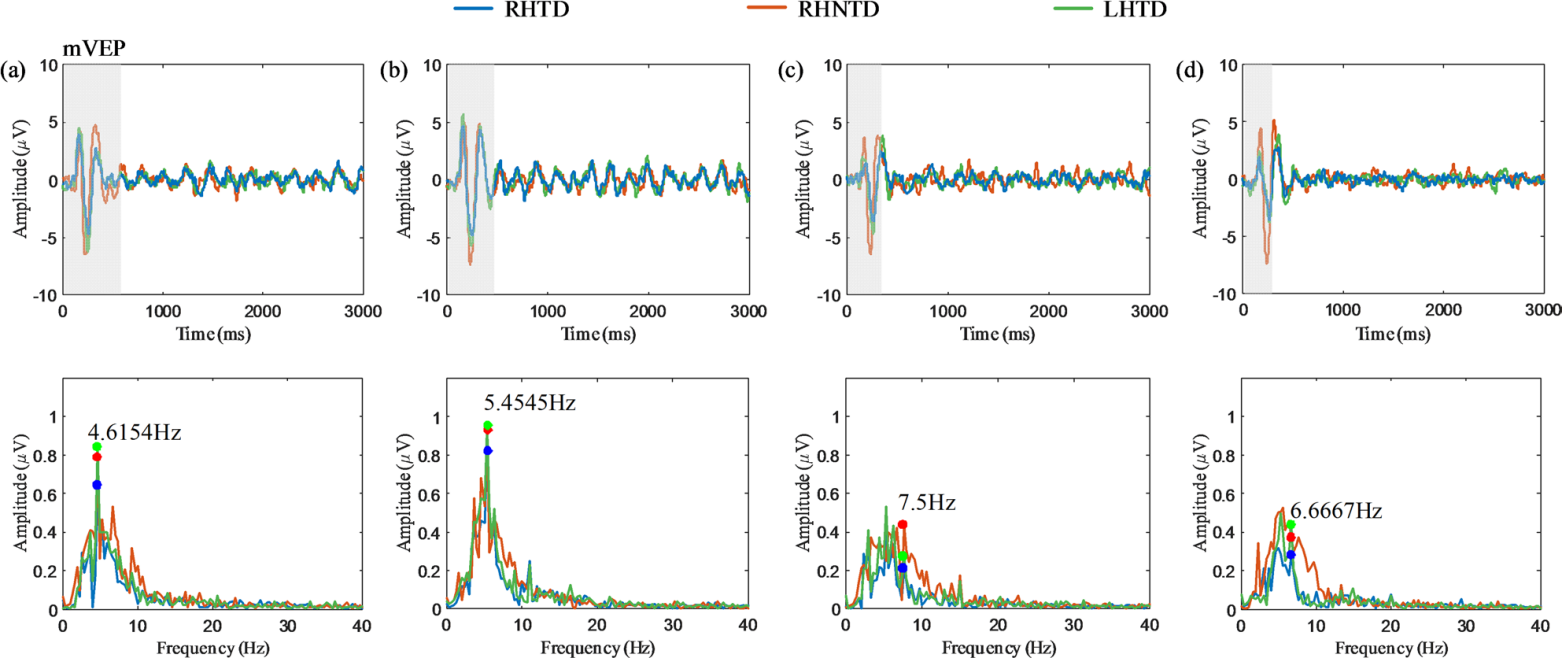
The average temporal domain waveforms and spectra of the Oz channel for all subjects induced by different stimulus targets in the three AO paradigms. **a** EEG response to Target 1 (grasping a paper cup/thumb adduction) **b** EEG response to Target 2 (grasping a small ball/grasping) **c** EEG response to Target 3 (pinching a dice/pinching with the thumb and index finger) **d** EEG response to Target 4 (tapping a mouse/bending the index finger)

This study employed the Oz channel to investigate the intensity of occipital cortex evoked features across different target frequencies. FFT spectral analysis was performed on EEG data from four stimulus frequencies across three AO paradigms during the 0–3 s stimulus period, with the spectral amplitude used as the feature. In Fig. 3, blue, red, and green denote the RHTD, RHNTD, and LHTD paradigm, respectively. The corresponding colored points indicate the amplitude at the response frequency. As shown in Fig. 3(a) and (b), the frequency-domain response of the Oz channel to AO stimuli exhibited significant spectral peaks at the corresponding stimulus frequencies. Notably, the amplitude at Target 2 is greater than those at the other frequencies. Additionally, typical mVEP transient components were observed in the time-domain waveforms of all four target frequencies: a positive deflection with a latency of 154–190 ms (P1) and a negative deflection with a latency of 220–270 ms (N2).

Significant differences in EEG signals were observed across AO paradigms at various target frequencies. A mixed-model ANOVA was conducted with stimulus target and paradigm type as fixed factors, participant as random factors, and Oz channel spectral amplitude at corresponding stimulus frequencies as the dependent variable. Regarding task-driven or not in AO paradigms, Fisher’s post hoc tests revealed significantly higher spectral amplitudes under the RHNTD paradigm than the RHTD paradigm at target 1 (*t* = 1.99, *p* = 0.048) and target 3 (*t* = 2.29, *p* = 0.023). Regarding the mirrored stimulus in AO paradigms, there was no significant difference on the amplitude at the stimulus frequencies between the RHTD paradigm and the LHTD paradigm.

### Analysis of sensorimotor cortex characteristics

**Fig. 4:**
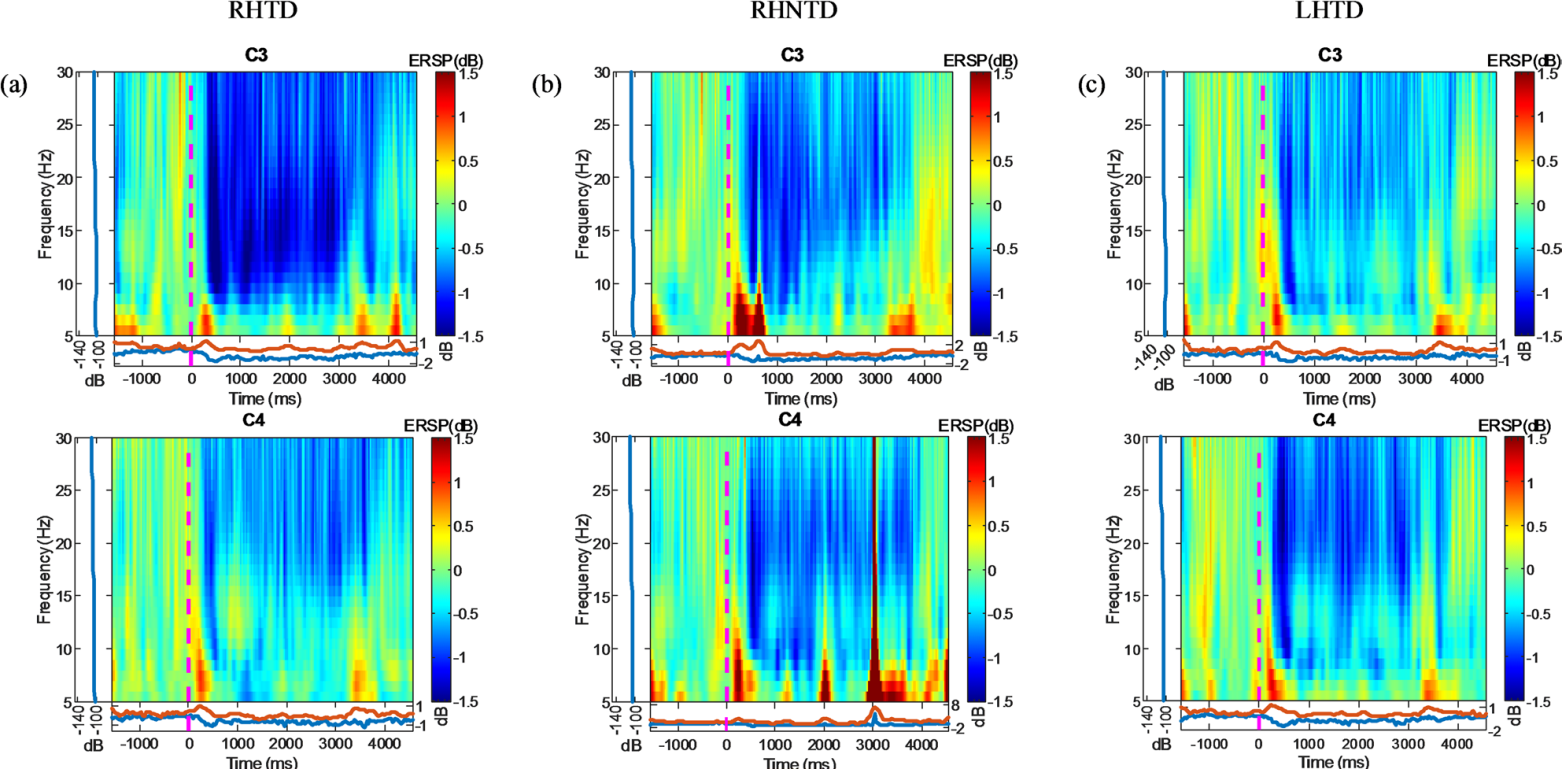
Average ERSP in channels C3 and C4 **a** Average ERSP during the RHTD paradigm **b** Average ERSP during the RHNTD paradigm **c** Average ERSP during the LHTD paradigm

This study investigated the differences in the ERSP between the C3 and C4 channels across three AO paradigms. Although data from multiple brain regions and frequency bands were acquired, the analysis focused on C3/C4 and the α/β bands due to their established relevance in sensorimotor activation during AO tasks [36–38]. As shown in Fig. 4, the ERD bilaterally in the primary sensorimotor cortex (C3/C4 electrodes), extending to the ipsilateral and contralateral sensorimotor regions. Three AO paradigms exhibited phase-locked ERD/ERS patterns: the separation of the ERD/ERS between channels C3 and C4 occurred within a few hundred milliseconds after stimulation onset, followed by the disappearance of ERS until it reappeared at 3000 ms, when stimulation ended, indicating the recovery of motor activity in the brain. ERD persisted until 4000 ms, with significantly stronger ERD from 0 to 3000 ms compared to other time intervals. In terms of the mirrored stimulus, as shown in Fig. 4(a) and (b), demonstrate stronger C3 ERD magnitudes than C4, which is concordant with right-hand AO task lateralization. Similarly, as shown in Fig. 4(c), for the left-hand AO task, the ERD magnitudes in the C4 channel are greater those that in the C3 channel. In terms of whether the AO paradigm is task-driven or not, as shown in the ERSP plots of the three AO paradigms, the RHTD AO paradigm shows the highest ERD amplitudes in the C3 channel.

**Fig. 5:**
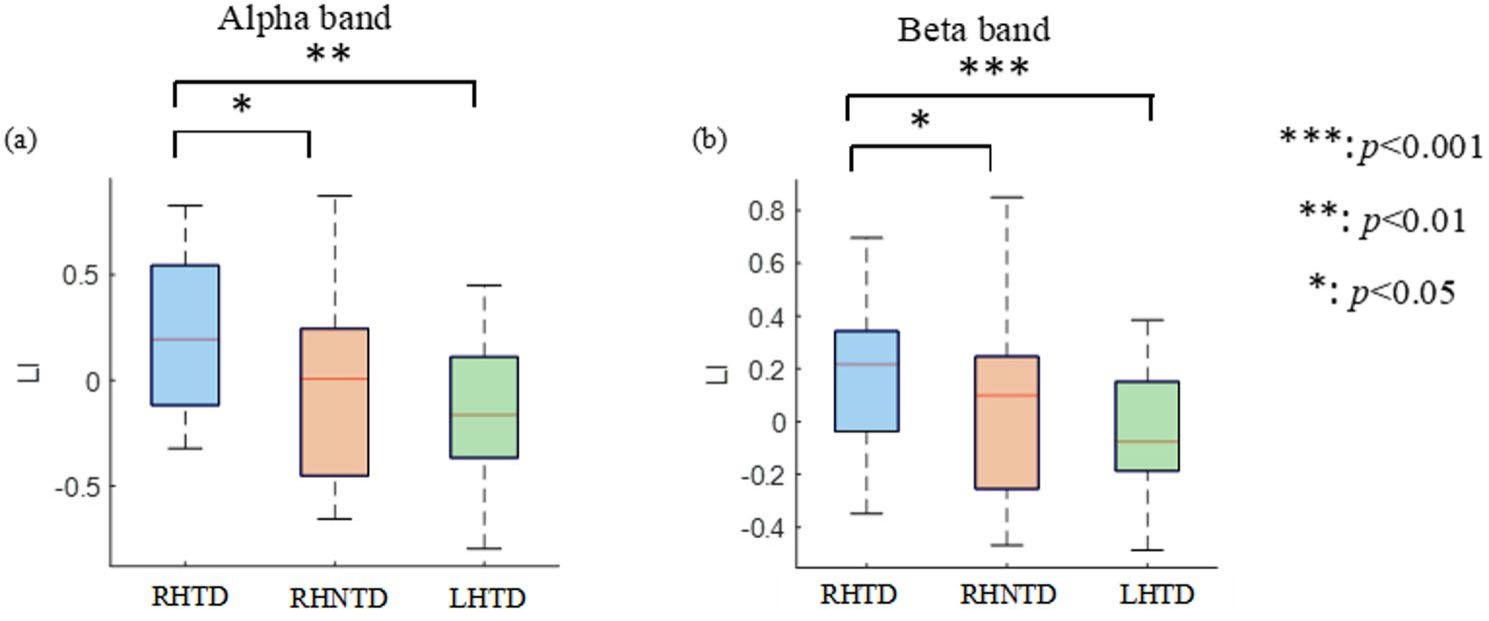
Compared the differences in the LI across different frequency bands under the three AO paradigms. **a** Differences in the LI in the α band **b** Differences in the LI in the β band

Figure 5 presents mixed-model ANOVA results comparing the LI values across the three AO paradigms in the α and β frequency bands. The model designated stimulus target and paradigm type were set as fixed factors, the participants were set as random factors, the dependent variable was LI, and Fisher’s post hoc tests was used to examine significant effects. In the α band, the LI for the RHTD paradigm was significantly higher than that for the RHNTD condition (*t* = -2.1, *p* = 0.037) and the LHTD condition (*t* = -3.10, *p* = 0.004). Similar results were observed in the β band, where the LI for the RHTD paradigm was significantly higher than that for the RHNTD paradigm (*t* = -2.11, *p* = 0.042) and the LHTD paradigm (*t* = -4.04, *p* ≤ 0.001).

### Analysis of the recognition accuracy utilized the TDCA Method

**Fig. 6:**
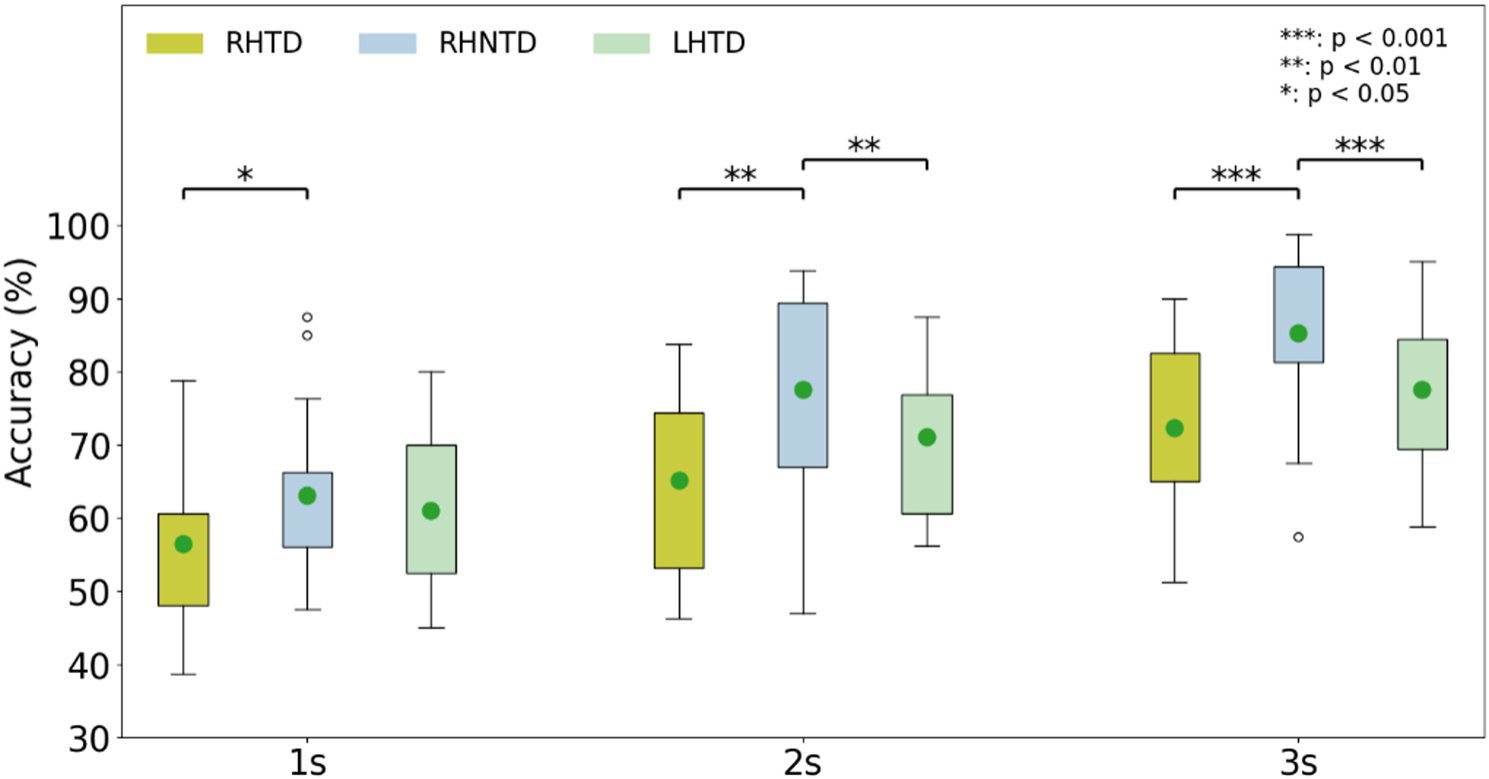
Comparison of the recognition accuracy of three AO paradigms under different data lengths. (Green dots indicate the mean values)

In this study, the recognition performance of the three designed AO paradigms was compared. And the EEG data lengths of 3 s, 2 s, and 1 s were utilized to investigate the impact of data length on recognition accuracy. On the whole, with the increase of data length, the recognition accuracy increased for each AO paradigm as shown in Fig. 6. Furthermore, the RHNTD paradigm achieved the highest recognition accuracy at 85.20% ± 10.97% with 3 s data length. While the RHTD paradigm revealed the lowest recognition accuracy for different data lengths.

The mixed-model ANOVA was utilized to analyze the significance. The paradigm type and the data length were set as fixed factors, the participants were set as random factors and the dependent variable was the recognition accuracy. Results showed that the paradigm type (*F* = 11.86, *p* < 0.001) and the data length (*F* = 58.79, *p* < 0.001) significantly affected recognition accuracy. In terms of whether the AO paradigm is task-driven or not, Post-hoc analysis showed that the recognition accuracies under the RHNTD paradigm were significantly higher than the recognition accuracies under the RHTD paradigm (3 s: *t* = 4.434, *p* < 0.001; 2 s: *t* = 3.522, *p* = 0.0024; 1 s: *t* = 2.459, *p* = 0.0243) and the LHTD paradigm (3 s: *t* = 4.035, *p* < 0.001; 2 s: *t* = 2.929, *p* = 0.009). With respect to the mirrored stimulus in AO paradigm, there was no significant difference on the recognition accuracies between the RHTD paradigm and the LHTD paradigm (3 s: *t* = -1.666, *p* = 0.1131; 2 s: *t* = -1.952, *p* = 0.067; 1 s: *t* = -1.379, *p* = 0.1849).

**Fig. 7:**
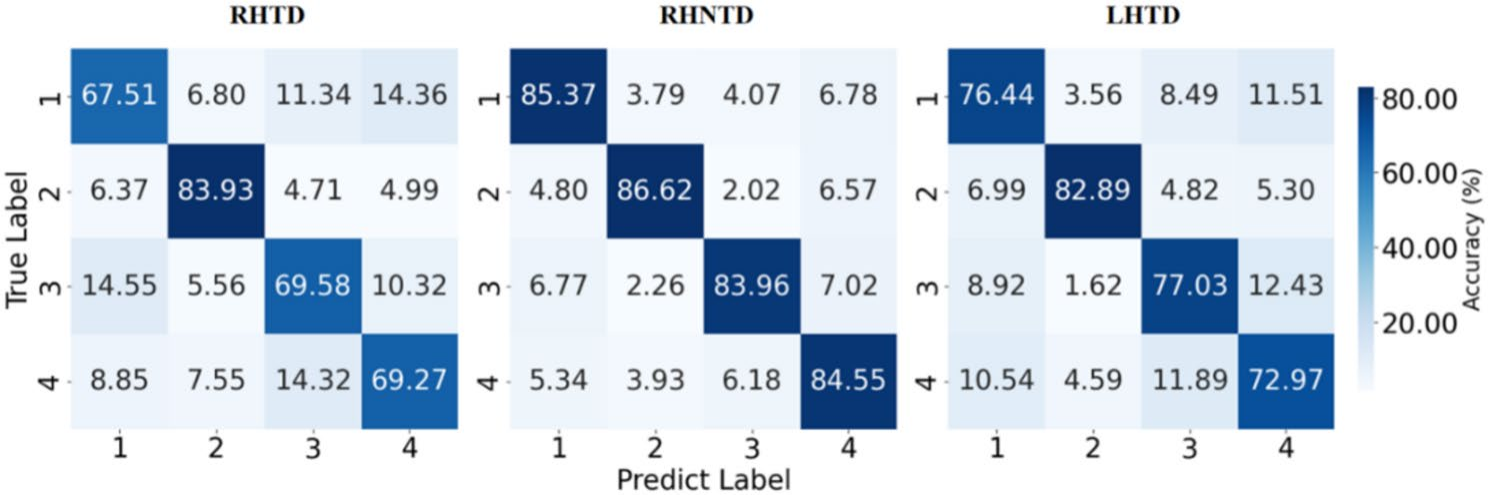
Heatmap of decoding confusion matrices for three AO paradigms within 3 s data length (The label 1, 2, 3, and 4 refer to target 1, 2, 3, and 4, respectively)

To further compare the recognition accuracy within 3 s of data, confusion matrix heatmaps were generated for the three AO paradigms. Figure 7 demonstrates demonstrate balanced and robust recognition accuracy for the four targets (accuracy > 83% for each target) in the RHNTD paradigm. The label 1, 2, 3, and 4 in Fig. 7 refer to the target 1, 2, 3, and 4 as shown in Fig. 1(a), respectively. Specifically, the target 1 refers to the AO stimulus of grasping a paper cup/thumb adduction. The target 2 refers to the AO stimulus of grasping a small ball/grasping. The target 3 refers to the AO stimulus of pinching a dice/pinching with the thumb and index finger. And the target 4 refers to the AO stimulus of tapping a mouse/bending the index finger. However, only target 2 showed good recognition accuracy both in the RHTD paradigm and the LHTD paradigm. Notably, the misclassification patterns for targets 1, 2, and 4 in the RHTD paradigm and the LHTD paradigm were consistent (e.g., the error rates of misidentifying target 1 as other targets, from highest to lowest, are as follows: target 4, target 3 and target 2). The misclassification patterns for targets 1, 3, and 4 in the RHNTD and LHTD paradigms were also consistent, and the misclassification patterns for actions 1 and 4 in the RHTD and RHNTD paradigms were consistent.

### The recognition accuracy crossing AO paradigms

**Fig. 8:**
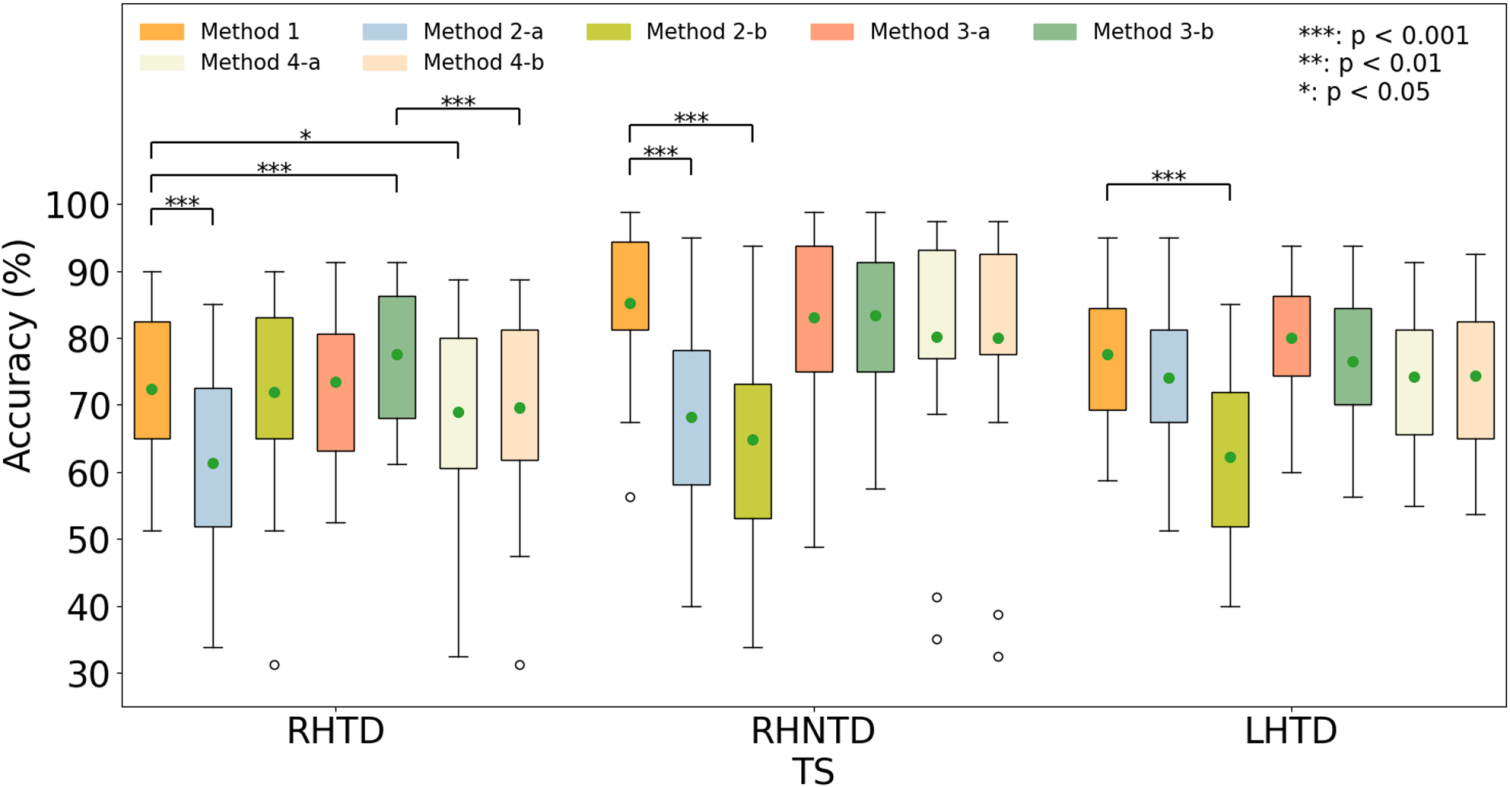
Comparison of cross-AO paradigm decoding. The [-a, -b] following the Method2/3/4 encode different SS paradigms in the three AO paradigms, respectively [RHNTD, LHTD], [RHTD, LHTD], and [RHTD, RHNTD]. The green dots represent the mean values

The recognition accuracy crossing AO paradigms was assessed as shown in Fig. 8. Several methods were designed to explore the recognition accuracy crossing AO paradigms. As shown in the Fig. 2, the Method 1 employed the EEG data from the TS paradigm for training, the Method 2 utilized the data from the SS paradigm for training, the Method 3 combined that data from SS and TS paradigms through naive transfer learning to construct training data, the Method 4 incorporated LST to transfer the data from the SS paradigm and jointly constructs training data with the data from the TS paradigm. The data from the TS paradigm were the test data in all methods. The [-a, -b] following the Method2/3/4 in Fig. 8 indicated utilizing the data from different SS paradigms, i.e., [RHNTD, LHTD], [RHTD, LHTD], and [RHTD, RHNTD], to construct the spatial filter *W* when the TS paradigm was RHTD, RHNTD, and LHTD, respectively. For instance, when the TS paradigm was RHTD, the training data to construct the spatial filter in the Method2-a were derived from RHNTD and the training data in the Method2-b were derived from LHTD. The current study analyzed the recognition accuracy crossing AO paradigms in two dimensions, i.e., (1) crossing the mirrored stimulus (left versus right-handed movements) and (2) crossing the task-driven actions in the AO paradigms (task-driven vs. non-task-driven).

With respect to crossing the mirrored stimulus, a comparison of the recognition accuracy between Method1 and Method2 revealed that using the EEG data under the RHTD paradigm and the LHTD paradigm interchangeably as training sets caused no significant changes in the recognition accuracy (Method1 versus Method2-b when the TS was RHTD, *p* = 0.9152; Method1 verse Method2-a when TS was LHTD, *p* = 0.3899). These results suggest that EEG data from these two paradigms can be directly used for cross-paradigm decoding. Specifically, combining the EEG data from the LHTD paradigm with the TS data significantly enhanced the recognition accuracy in the RHTD paradigm, i.e., from 72.37% ± 11.54% (Method 1) to 77.50% ± 10.04% (Method3-b) (*p* < 0.001). Similarly, combining the EEG data from the RHTD paradigm with the TS data also improved recognition accuracy in the LHTD paradigm, i.e., from 77.50% ± 10.84% (Method1) to 80.00% ± 9.07% (Method3-a).

In terms of crossing the task-driven actions in the AO paradigms, interchanging the training data from the RHNTD paradigm with either from the RHTD paradigm or the LHTD paradigm significantly reduced the recognition accuracy (Method1 verse Method2-a when the TS was RHTD, Method1 verse Method2-a when TS was RHNTD, Method1 verse Method2-b when the TS was RHNTD, Method1 verse Method2-b when TS was LHTD, all *p* < 0.001). Moreover, combining the EEG data from the RHTD paradigm or the LHTD paradigm with the TS data couldn’t improve the recognition accuracy in RHNTD paradigm (Method3-a or Method3-b versus Method1 when TS was RHNTD). These results suggest that EEG data from observing the AO paradigm, whether task-driven or not, were directly used for training or combed with TS data, the recognition accuracy decreased.

In addition, the LST was utilized to project the SS data into the TS distribution. Comparing Method3 and Method4, no significant improvement in recognition accuracy was observed with the LST, with instances of a significant degradation in performance between Method3-b and Method4-b when the TS was RHTD.

Finally, conducting experiments with the soft robotic glove feedback in AO-based BCI served two purposes. First, to demonstrate that we have developed a BCI system intended for motor function rehabilitation. More importantly, we aimed to simulate real-world usage scenarios. Given that EEG data under some AO paradigms already exist, can this existing EEG data be utilized for AO-based BCI training targeting other actions? Building upon prior results, we further validated the proposed method3 proposed in the current study.

For the RHTD paradigm with the action feedback, we utilized the Method 3 to construct spatial filters by respectively concatenating one run’s data from the RHTD paradigm (Action Feedback) with four runs’ EEG data from the RHTD, RHNTD, and LHTD paradigms (Visual Feedback). We then compared the recognition accuracy under three different training data conditions. We implemented a two-fold cross-validation process matching the experimental conditions. For the LHTD paradigm with the action feedback, the same data processing method was applied.

**Fig. 9:**
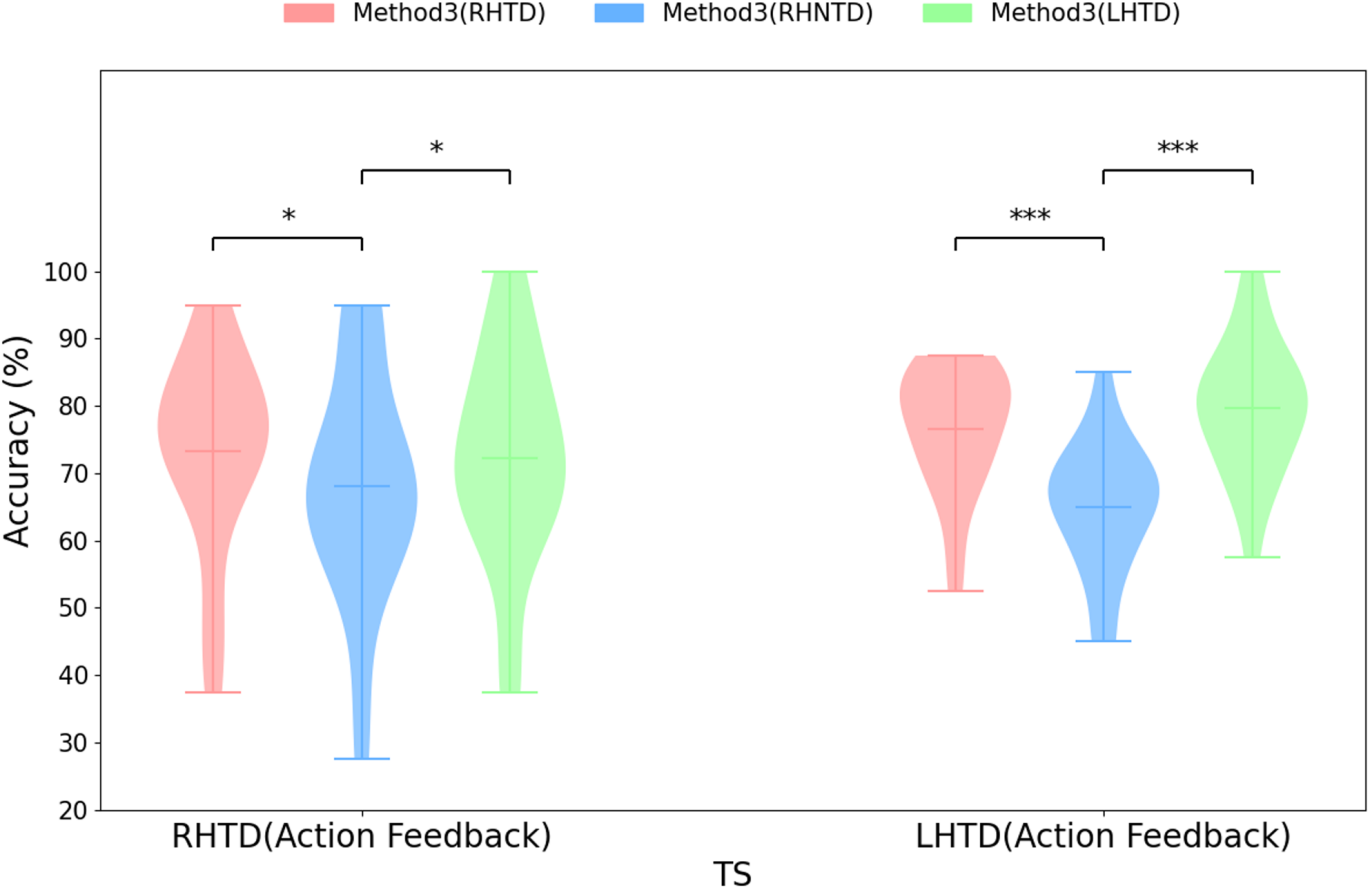
Comparison of the recognition accuracy under the action feedback condition in the RHTD paradigm and the LHTD paradigm utilizing the designed Method3 within different training data (EEG data from different AO paradigm)

As shown in Fig. 9, using the EEG data from the RHTD (Action Feedback) condition as the TS, the average recognition accuracy within different training data achieved 73.37 ± 14.69% (Method3(RHTD)), 68.16 ± 17.20% (Method3(RHNTD)), and 72.21 ± 15.84%(Method3(LHTD)), respectively. Statistical tests indicated that the recognition accuracy of the Method3(RHTD) was significantly higher than that of the Method3(RHNTD) (*t* = 2.6495, *p* = 0.0163). While no significant difference in the recognition accuracy was observed between the Method3(RHTD) and the Method3(LHTD) (*t* = 0.8002, *p* = 0.4340).

Similarly, using the EEG data from the LHTD (Action Feedback) condition as the TS, the average recognition accuracy within different training data achieved 76.55 ± 9.42% (Method3(RHTD)), 64.92 ± 9.95%(Method3(RHNTD)), and 79.63 ± 10.09%(Method3(LHTD)), respectively. Statistical analysis revealed that the recognition accuracy of the Method3(LHTD) significantly higher than that of the Method3(RHNTD) (*t* = 7.0040, *p* < 0.001). While no significant difference in the recognition accuracy was observed between the Method3(LHTD) and the Method3(RHTD) (*t* = 2.0762, *p* = 0.0525).

## Discussion

To the best of our knowledge, this is the first study on cross-AO paradigms decoding for AO-BCI. The main results of this study are as follows: (1) The three designed AO paradigms could elicit SSMVEP in the occipital cortex and induce SMR in the sensorimotor cortex. (2) The RHTD paradigm evoked stronger ERD and LI. (3) The RHNTD paradigm achieved the highest recognition accuracy at 85.20% compared with the RHTD paradigm (72.37%) and the LHTD paradigm (77.50%). (4) The online feedback in AO-BCI provided by visual feedback or the movement feedback provided by the soft robotic glove have no significant effects on the recognition accuracy. (5) When combing EEG from the mirrored stimulus and TS data, the recognition accuracy can be further improved to 77.50% (RHTD) and 80% (LHTD) respectively.

This study quantified occipital cortex activation patterns induced by three AO paradigms through EEG feature extraction. In the frequency domain analysis of the occipital cortex (Fig. 3), all paradigms exhibited significant spectral peaks at the stimulus frequencies corresponding to target 1 (grasping a paper cup/thumb adduction) and target 2 (grasping a small ball/grasping), indicating that the brain revealed selective neural responses to AO stimuli at specific frequencies. Transient components of the mVEP were observed in the time domain waveforms, suggesting that AO tasks dynamically modulate neural activity in the visual pathway. These results indicate that the three AO paradigms induced SSMVEP in the occipital cortex. Further statistical analysis of the three AO paradigms revealed significant differences between the RHTD and RHNTD paradigms in both target 1 (grasping a paper cup/thumb adduction) and target 3 (pinching a dice/pinching with the thumb and index finger). Current research indicates that, among the three AO paradigms, the RHNTD paradigm exhibit stronger activation characteristics in the occipital cortex. This may be due to the non-task-driven paradigm reducing the cognitive load, thereby concentrating visual attention resources on the dynamic features of target stimulus movements [39].

Additionally, this study extracted and analyzed features from the sensorimotor cortex, and revealed that ERD was significantly stronger during the stimulus phase, i.e., from 0s to 3s, than during other time intervals across the three AO paradigms, indicating that the paradigms significantly activated the sensorimotor cortex. The significantly greater C3 ERD intensity than C4 intensity in the RHTD/RHNTD paradigms reflected the specific activation of the left hemisphere sensorimotor cortex. Which aligns with the motor preparation and execution requirements of the right-hand task. Therefore, in the LHTD AO paradigm, the ERD in the C4 channel was higher. On the basis of the above phenomena, the results of this study are consistent with the fMRI research by Ward et al., demonstrating enhanced blood oxygen level signals in the contralateral sensorimotor cortex [40], supporting ERD as a sensitive indicator of neuronal activation. A comparison of the three AO paradigms revealed that the ERD activation level was highest in the RHTD paradigm, indicating that task-driven paradigms can more strongly activate the MNS.

Further statistical analysis of the LI across the three AO paradigms revealed that LI analysis demonstrated positive values for the RHTD/RHNTD paradigms but negative values for the LHTD paradigm, reflecting the neural mechanism where unilateral movement is dominated by the contralateral hemisphere [41]. Additionally, significant LI differences emerged among the paradigms in both α and β bands. The LI results for the RHTD paradigm were significantly higher than those for the other two AO paradigms, consistent with the ERSP topography. The above experimental results indicate that the AO paradigm used in this study can help activate the SMR of the motor area. Among these, the RHTD paradigm significantly activates ERD more than other paradigms do, indicating that task-driven AO using the dominant hand can optimally activate the sensorimotor cortex. Comparing the RHTD paradigm with the RHNTD paradigm, task-driven paradigms enhance sensorimotor cortex lateralization responses, which is consistent with previous research findings.

Further analysis of the recognition accuracy of the three AO paradigms in the occipital cortex revealed that the content of the AO paradigms significantly influenced the recognition accuracy of the AO-BCI system under different data lengths. Additionally, the recognition accuracy of the RHNTD paradigm was superior to that of the RHTD and LHTD paradigms, which is consistent with the conclusion that the RHNTD paradigm elicits stronger activation features in the occipital cortex. The recognition accuracy of the RHTD paradigm and the LHTD paradigm was not good, and the activation features in the occipital cortex were weak. Under the RHTD paradigm or the LHTD paradigm, the participants’ attention might be focused on the task objects (the cup, the dice, the ball, and the mouse) rather than the hand movements themselves. It might be the task objects in the RHTD paradigm or the LHTD paradigm that causing the significantly lower recognition accuracy compared with the RHNTD paradigm. The participants also reported such content confound issues during the experiment. Strauss DJ et al. also noted that autonomous attention is not a sufficient condition for immersion [42].

In addition, the decoding in current study is occipital driven, and the reported accuracies primarily reflect stimulus locked visual responses. A recent study showed an average accuracy of 43.61% for the MI task in the 4-finger classification utilizing the EEG data from the sensorimotor cortex [43]. Cross-domain decoding accuracy in the motor cortex may further decline to the chance level. Thus, the current study did not try the motor-based decoding.

Research on cross-AO paradigms decoding indicates that training solely on SS-paradigm data for direct decoding of TS-paradigm data may lead to reduced recognition accuracy. To maximize information utilization under lower practical constraints, the full SS training data was employed. In the naive transfer learning scheme (Method3), analysis of the recognition accuracy of combined features across paradigms regrettably revealed that RHTD and LHTD paradigms decoding did not improve RHNTD paradigm decoding. This implies that more appropriate data calibration methods are needed to achieve auxiliary decoding for RHNTD paradigm decoding. However, this study revealed that the RHTD and LHTD paradigms presented significant cross-paradigm compatibility. Under limited data conditions, better recognition accuracy can be achieved through simple concatenation (naive transfer learning, Method3). Concurrently, guided by the theory of cross-limb neural compensation [19–21], rehabilitation of the affected hand could be further enhanced. This study demonstrates that within the AO-BCI framework the mirrored stimulus variants can mutually enhance the recognition accuracy. This discovery offers a fresh perspective on optimizing BCI performance. In addition to refining algorithms and single paradigms, designing appropriate mirrored stimulus also represents an effective pathway for enhancing accuracy. In addition, no significant difference in the recognition accuracy between the Method3(LHTD) and the Method3(RHTD) were founded as shown in Fig. 9. Thus, the mirrored stimulus, i.e., the RHTD paradigm and the LHTD paradigm, were compatible by utilized the designed Method3. When there was only a small amount of TS data (e.g. one run’s data), the recognition accuracy can be improved by using Method 3 to combine data in the mirrored stimulus.

Additionally, the LST-based method achieved significantly higher decoding accuracy than the standard task-related component analysis (TRCA)-based method and the non-LST naïve transfer-learning method [35]. While the current study utilized the TDCA method which was outperformed TRCA method [26]. Thus, the designed naïve transfer-learning (Method3) in the current study had achieved a relatively high recognition accuracy. The role of the LST to further improve the recognition accuracy might be not obvious.

## Limitations

This study has several limitations. First, considering participant engagement and experimental duration, the left-hand no-task-driven paradigm was omitted from the current study. Second, given the current low accuracy of decoding fine motor movements on the basis of the sensorimotor cortex, this study focuses on cross-paradigm transfer decoding utilizing features from the occipital cortex, rather than conducting analyses targeting the sensorimotor areas. Our future work will explore the fusion algorithm that integrated MI and SSMVEP features induced by the AO paradigm. Especially for subjects who show low decoding accuracy based on SSMVEP but high accuracy based on ERD. Finally, the current study analyzes cross-paradigm decoding in AO-BCI, with the goal of assessing its feasibility and performance. In addition, the current only recruited the healthy participants. There may be fundamental differences between healthy and post-stroke brains. Developing algorithms to further improve the recognition accuracy and accessing transferable cross-paradigm decoding in patients will be our next study.

## Conclusion

This study demonstrated that the three designed AO paradigms could effectively elicit both SSMVEP and SMR responses, their activation patterns exhibited distinct paradigm specificity. And the RHNTD paradigm achieved the highest recognition accuracy at 85.20% ± 10.97%. In terms of the mirrored stimulus in the AO paradigm, EEG data from the occipital cortex can be directly utilized in cross-paradigm training to increase recognition accuracy. In terms of whether the AO paradigm is task-driven or not, the task-driven paradigm can produce stronger ERD and lateralization in sensorimotor areas. Due to the content confound in the task-driven AO paradigm, the EEG data require further development of calibration methods to be effectively applied in cross-paradigm decoding.

## Data Availability

Data will be made available on request.

## Abbreviations

BCI: Brain-computer interface
AO: Action observation
AO-BCI: Action observation brain-computer interface
MNS: Mirror neuron system
EEG: Electroencephalogram
MI: Motor imagery
ERD: Event-related desynchronization
SSMVEP: Steady-state motion visual evoked potential
SMR: Sensory motor rhythm
TDCA: Task discrimination component analysis
TS: Target-session
SS: Source-session
RHTD: Right-hand task-driven
RHNTD: Right-hand no-task-driven
LHTD: Left-hand task-driven
ERSP: Event-related spectral power
LI: Laterality index
LST: Least squares transformation
